# Intravitreal allogeneic mesenchymal stem cells: a non-randomized phase II clinical trial for acute non-arteritic optic neuropathy

**DOI:** 10.1186/s13287-023-03500-7

**Published:** 2023-09-21

**Authors:** Jose C. Pastor, Salvador Pastor-Idoate, Marina López-Paniagua, Marta Para, Francisco Blazquez, Esther Murgui, Verónica García, Rosa M. Coco-Martín

**Affiliations:** 1 Instituto de Oftalmobiología Aplicada (IOBA), Universidad de Valladolid, Campus Miguel Delibes, Pº de Belén nº 17, 47011 Valladolid, Spain; 2 Centro en Red de Medicina Regenerativa y Terapia Celular de Castilla y León, Valladolid, Spain; 3 Redes de Investigación Cooperativa Orientadas a Resultados en Salud (RICORS), Instituto de Salud Carlos III, Madrid, Spain; 4 Hospital Clínico Universitario, Valladolid, Spain; 5 Centro de Investigación Biomédica en Red de Bioingeniería, Biomateriales y Nanomedicina (CIBER-BBN), Instituto de Salud Carlos III, Madrid, Spain; 6 Unidad de Excelencia Instituto de Biología y Genética Molecular (IBGM), Universidad de Valladolid-CSIC, Valladolid, Spain; 7 Citospin S.L., Valladolid, Spain

**Keywords:** NA-AION, Acute anterior ischemic optic neuropathy, MSV®, Allogeneic bone marrow-derived mesenchymal stem cells, BM-MSCs, Bone marrow mesenchymal stem cells

## Abstract

**Background:**

An effective treatment for acute non-arteritic ischemic optic neuropathy (NA-AION) has not been known or proven yet. Previous studies have suggested a neuroprotective effect of allogeneic bone marrow-derived mesenchymal stem cells. This study aims to report the results of a clinical trial on patients with acute non-arteritic optic neuropathy (NA-AION) treated with an intravitreal injection of allogeneic bone marrow-derived mesenchymal stem cells (BM-MSCs) (MSV®).

**Methods:**

We conducted a prospective, non-randomized, clinical phase-II study (Eudra CT number 2016-003029-40; ClinicalTrials.gov Registry NCT03173638) that included 5 patients with acute unilateral NA-AION diagnosed within 2 weeks after symptom onset and who received an intravitreal injection of allogeneic BM-MSCs (0.05 ml; cell concentration: 1.5 × 10^6^cells/mL). The patients underwent regular ophthalmological examinations and were followed for one year.

**Results:**

In this trial, allogeneic BM-MSCs appeared to be safe as no patients developed signs of acute nor chronic intraocular inflammation or a significant change in intraocular pressure, although an epiretinal membrane was developed in one patient. A retrolental aggregate formed shortly after the injection spontaneously disappeared within a few weeks in another phakic patient, leaving a subcapsular cataract. Visual improvement was noted in 4 patients, and amplitudes of P100 on the visually evoked potentials recordings increased in three patients. The retinal nerve fiber layer and macular ganglion cell layer thicknesses significantly decreased during the follow-up.

**Conclusions:**

Besides the development of an epiretinal membrane in one patient, the intravitreal application of allogeneic BM-MSCs appeared to be intraocularly well tolerated. Consequently, not only NA-AION but also BM-MSCs deserve more clinical investigational resources and a larger randomized multicenter trial that would provide stronger evidence both about safety and the potential therapeutic efficacy of intravitreally injected allogeneic BM-MSCs in acute NA-AION.

*Trial registration*: Safety Assessment of Intravitreal Mesenchymal Stem Cells for Acute Non-Arteritic Anterior Ischemic Optic Neuropathy (NEUROSTEM). NCT03173638. Registered June 02, 2017 https://clinicaltrials.gov/ct2/show/NCT03173638.

**Supplementary Information:**

The online version contains supplementary material available at 10.1186/s13287-023-03500-7.

## Introduction

Non-arteritic anterior ischemic optic neuropathy (NA-AION), the most common ischemic optic neuropathy [1, 2], usually involves classical symptoms and signs that lead easily to diagnosis. NA-AION is mostly a disease of middle-aged and elderly individuals. Several risk factors have been identified, such as diabetes, small cup-to-disk ratio or crowded disk, hyperlipidemia, systemic hypertension, nocturnal hypotension, sleep apnea, and others [1, 2]. Patients generally describe sudden and painless visual deterioration, mostly noticed on morning awakening, and often complain of severe visual loss, severe visual field defects, and an ipsilateral relative afferent pupillary defect (RAPD) [1, 2]. The most important clinical outcome on ophthalmic analysis at the beginning of visual loss is optic disk edema that improve spontaneously in some weeks, leading in generalized or sectoral atrophy of the optic disk [1, 2]. The presence of a few splinter hemorrhages on the optic disk or peripapillary region is also a commonly associated finding [1, 2]. Despite extensive studies, the etiology of NA-AION is not known definitively, but the best evidence suggests the cause is an infarction in the region of the optic nerve head (ONH), which is perfused by short posterior ciliary arteries and their branches with a relatively low perfusion pressure [3]. The Ischemic Optic Neuropathy Decompression Trial described its natural history, i.e., about 30% of patients regain three or more lines of vision, 20% lose three or more lines of vision, and most patients have an unchanged vision at 2 years of follow-up. However, it is assumed that visual acuity (VA) does not change in most patients after the resolution of the acute event and that the patients who gain a few lines of vision likely learned to improve their residual fixation [4].

Despite increasing knowledge about the risk factors and clinical findings of NA-AION, there is no effective treatment, and the existing ones lack a clear evidence-based benefit [5, 6].

Because there is great interest in neuroprotective therapy for ischemic stroke and various types of optic neuropathies, this approach has been suggested for NA-AION. However, a recent review found that despite all the experimental and clinical research on neuroprotective agents in NA-AION, no scientific evidence shows that any of the suggested molecules had any beneficial effect in human clinical studies [7].

In 2017, the Stem Cell Ophthalmology Treatment Study (SCOTS) reported 10 patients with bilateral visual loss due to NA-AION who was treated with autologous bone marrow-derived stem cell (BMSC) therapy and achieved visual improvements [8]. The authors suggested that proteins and hormones with paracrine effect secreted by BMSC as well as the secretion of microvesicles or exosomes loaded with messenger RNA or other compounds could mediate visual improvement in patients. Also, these authors hypothesized that other mechanisms as differentiation of BMSC to neural cells and/or transfer of mitochondria could be involved in this outcome. However, although the authors claimed that some patients had clear improvement, the study had several methodological weaknesses as they included, for example, many different diseases and routes of administration that affect the robustness of the published results [8]. By 2017, we analyzed the feasibility, safety, and biocompatibility of intravitreal injection of human bone marrow mesenchymal stem cells (BM-MSCs) expanded under Good Manufacturing Practice in immunocompetent pigmented rabbits that tolerated the dose of 15 × 10^6^ cells/ml. Specifically the MSV®, Investigational Product (IP) 15–007, was used [9].

MSCs display significant anti-proliferative, anti-inflammatory, and anti-apoptotic features in the neural environment and until now, 20 clinical trials of MSC transplantation have been performed in patients mainly after ischemic stroke. We focused our study on NA-AION because the optic nerve is considered a part of the central nervous system [10, 11]. We used allogeneic bone marrow-derived expanded MSCs due to their numerous advantages over autologous ones and the absence of immune rejection by allogeneic MSC transplantation [12–14], as these cells express moderate quantity of human leukocyte antigen (HLA) major histocompatibility complex (MHC) class I and do not express Human Leukocyte Antigens—DR isotype (HLA-DR, MHC II) unless specific stimulation, and the classic co-stimulatory molecules [15, 16].

We also have extensive experience with the use of these MSV® cells in patients with limbal stem cell deficiency [17]. Furthermore, the concept of a therapeutic window is relatively well defined in ischemic stroke, and current treatments aimed at restoring cerebral blood flow are applied within a narrow timeframe to prevent further damage at the penumbra area that surrounds the infarct core and where some neurons have not yet undergone irreversible changes [18]. In this clinical trial (CT), we have applied these concepts.

The purpose of this work was to describe the results of intravitreal injection of allogeneic BM-MSCs (MSV®) in five patients with acute NA-AION who had been followed for a period of 12 months.

## Materials and methods

The Clinical Research Ethics Committee of the Valladolid East Health Area and the Spanish Agency for Medicine and Medical Devices (AEMPS) approved the study protocol, which followed European laws and the Declaration of Helsinki with its subsequent amendments. The Eudra CT number is 2016-003029-40 and the ClinicalTrials.gov Registry number is NCT03173638. Written consent was obtained from each patient prior to their participation in the study.

### Study design

The study was a prospective, non-randomized, phase II CT to determine the safety of mesenchymal stem cells intravitreally injected into patients with acute NA-AION. (see online supplementary figure S1). 

### Patients

Patients who presented with acute NA-AION within the first 2 weeks after symptom onset and met the inclusion and exclusion criteria were included after providing written informed consent.

The inclusion criteria included patients with acute unilateral NA-AION who presented within the first 2 weeks after symptom onset. NA-AION was defined by at least two of the following clinical characteristics: sudden painless loss of monocular vision; ONH edema; a clear RAPD; patients age of 50 years or older; and the ability to freely provide informed consent and complete the data protection form for study participation.

The general exclusion criteria included a medical history, erythrocyte sedimentation rate (ESR), and C-reactive protein (CRP) values compatible with a diagnosis of giant cell arteritis; evidence of any other etiology justifying optic neuropathy, even in the contralateral eye; a history of systemic vasculitis, multiple sclerosis, collagen disease, or cancer treatments; a positive pregnancy test at baseline in fertile women (for this purpose, menopause of at least 1 year from the baseline visit, bilateral oophorectomy, and/or total hysterectomy with adnexectomy indicated that women were not fertile); hypersensitivity or allergy to any of the active ingredients or excipients of an advanced therapy investigational medicinal product; and participation in any other CT with drugs or diagnostic or therapeutic instruments in the 2 months before inclusion in this study.

The ophthalmologic exclusion criteria included a history of uveitis or active eye inflammation; history or evidence of glaucoma or an intraocular pressure (IOP) of 24 mmHg or higher in either eye; media opacity hindering posterior pole examination; retinal pathologies in the affected eye; a history of cataract, vitreous, or glaucoma surgery during the previous 3 months in the affected eye.

### The source of cells and administration

Allogeneic human BM-MSCs (MSV®) cultured under Good Manufacturing Practice (PEI Number 15-007) and following the method for obtaining an enriched population of functional MSCs (Patent Number PCT/EP2019/074991) were used. Cells were provided the cell processing unit of the Institute of Biology and Molecular Genetic (IBGM, R&D Building of the Miguel Delibes Campus, Valladolid, Spain) that is accredited by the Spanish Agency of Medications and Medical Devices (AEMPS) (ES/102I/22 University of Valladolid-Citospin S.L.). Cells were characterized by fluid flow cytometry following the last update of the criteria of the International Society for Celle Therapy [19]. Cells were positive (expression ≥ 97%) for CD105, CD73, CD90, and CD166 markers and negative (expression ≤ 1%) for CD34 (hematopoietic stem cells and endothelial cells), CD45 (leukocytes and hematopoietic progenitors), CD14 (monocytes and macrophages), and HLA-DR (human leukocyte antigen: D related antigen, usually present in all cells and lymphocytes) markers. These results showed the presence of MSCs and the absence of other cell types of bone marrow in the advanced therapy product. In addition, our research group showed that cells secrete several cytokines and other trophic factors (as brain-derived neurotrophic factor (BDNF) and ciliary neurotrophic factor (CNTF)) that can potentially retard neuroretina degeneration by neuroprotective effect [20, 21]. We showed that the secretome obtained from these cells maintains neuroretinal morphology and decreases pro-apoptotic and pro-necroptotic gene and protein expression in neuroretina. In addition, these cells can regulate autophagy genes and proteins and promote antioxidant genes in the retina [22]. Cells were previously approved by the AEMPS and were used in other CT for several clinical indications, e.g., degenerative disk disease, disease [23], knee osteoarthritis [24], lupus nephritis [25], and limbal stem cell deficiency [17]. Patients were treated with MSV® obtained from different donors, except two patients that were treated with cells obtained from the same donor.

This investigational product is packaged in a 1-ml Luer lock syringe containing 150 µl of an isotonic medium with a cell concentration of 1.5 × 10^6^ cells/ml. Advanced therapy product showed the following requirements: (1) visual apparency as cellular pellet, (2) absence of mycoplasma (it was tested following the European Pharmacopoieia (Eur.Ph.) 2.6.7. using the Bact/Alert technology), (3) sterility (it was tested following the Eur. Ph.2.6.27 by PCR), (4) cell viability ≥ 93%, (5) number of cell duplications ≤ 5, and (6) specific marker expression (see cell characterization). A 50-µl (0.05 ml) suspension was injected using a 25G needle into the vitreous cavity via the pars plana 3.5 mm from the limbus in pseudophakic eyes and 4.5 mm from the limbus in phakic eyes after topical anesthesia using lidocaine 2% (Braun, Barcelona, Spain) in an operating room. After injection, a topical antibiotic (tobramycin 3 mg/ml, Tobrex, Alcon, Barcelona, Spain) was used 5 times daily for 5 days.

### Ophthalmic examination

The examination included measurement of the best-corrected visual acuity (BCVA) expressed in letters using the Early Treatment Diabetic Retinopathy Study test, IOP measured by Perkins tonometry, and examination of the anterior pole with a slit-lamp and of the posterior pole under pharmacologic mydriasis. Lens opacities were recorded according to LOCS III classification [26]. Color retinography and fundus autofluorescence images were obtained using the TRC 50DX type IA tool (Topcon Europe Medical BV, Capelle aan den Ijssel, The Netherlands) using Topcon IMAGEnet i-base version 3.14.4 software. The retinal nerve fiber layer (RNFL) and ganglion cell layer (GCL) thicknesses were assessed using the Cirrus 5000 spectral-domain optical coherence tomograph (SD-OCT) (Carl Zeiss Meditec Inc., Dublin, CA) and the Optic Disk Cube 200 × 200 and the Macular Cube 512 × 128 protocols, respectively, before and after intravitreal injection. Pattern-reversal visual-evoked potential (PRVEP) and flash visual-evoked potential (FVEP) recordings were evaluated with the computerized Optoelectronic Stimulator Vision Monitor MonPack 120 (Metrovision, Pérenchies, France), according to the International Society for Clinical Electrophysiology of Vision protocols. [27]

The clinical evaluation included one baseline visit, one PEI administration visit, and 6 months of follow-up visits over 12 months (see online supplementary table S2). The primary endpoint was safety, defined as the absence of ocular inflammation up to 12 months after treatment according to the Standardization of Uveitis Nomenclature for Reporting Clinical Data, i.e., reaction > 1+ in the anterior pole, the fibrinoid reaction in the anterior chamber, or reaction > 2+ in the vitreous [28].

## Results

Five patients (3 men, 2 women; age range, 59–85 years) were included; their demographic data are shown in Table 1. All had typical NA-AION characteristics including a sudden decrease in central vision (range, 0–25 letters), RAPD, and sectorial papillary edema with peripapillary hemorrhages. None had clinical suspicion of giant cell arteritis, having all normal ESR and CRP values.

**Table 1:** Demographic characteristics and BCVA data (Letters)

Patient	Age range (s)	Gender	Eye	Date of onset	Date of injection	Systemic risk factors	Basal BCVA	BCVA 1 Month	BCVA 3 Months	BCVA 6 Months	BCVA 12 Months	Lens status
1	80	Female	RE	25/11/2020	3/12/2020	HBP, cardiopathy	17	26	25	23	27	Cataract progression from C1N1 to C1N2PSC1
2	60	Male	LE	28/01/2021	8/2/2021	No	0	35	54	63	54	Cataract progression from C1N1 to C1N2PSC3
3*	70	Female	RE	8/03/2021	23/03/2021	HCh	24	33	1	2	0	Pseudophakia needed
4	60	Female	RE	19/02/2021	8/03/2021	HBP, HCh	25	73	80	80	67	Cataract progression from C1N1 to C2N2PSC1
5	50	Male	RE	15/03/2021	29/03/2021	HBP	14	36	51	48	57	Pseudophakic

*RE* right eye, *LE* left eye, *BCVA* best-corrected visual acuity, *HBP* high blood pressure, *HCh* hypercholesterolemia, *m* meter(s), *C* cortical, *N* nuclear, *PSC* posterior subcapsular

*Patient developed an epiretinal membrane + cataract needing phacovitrectomy

**Fig. 1:**
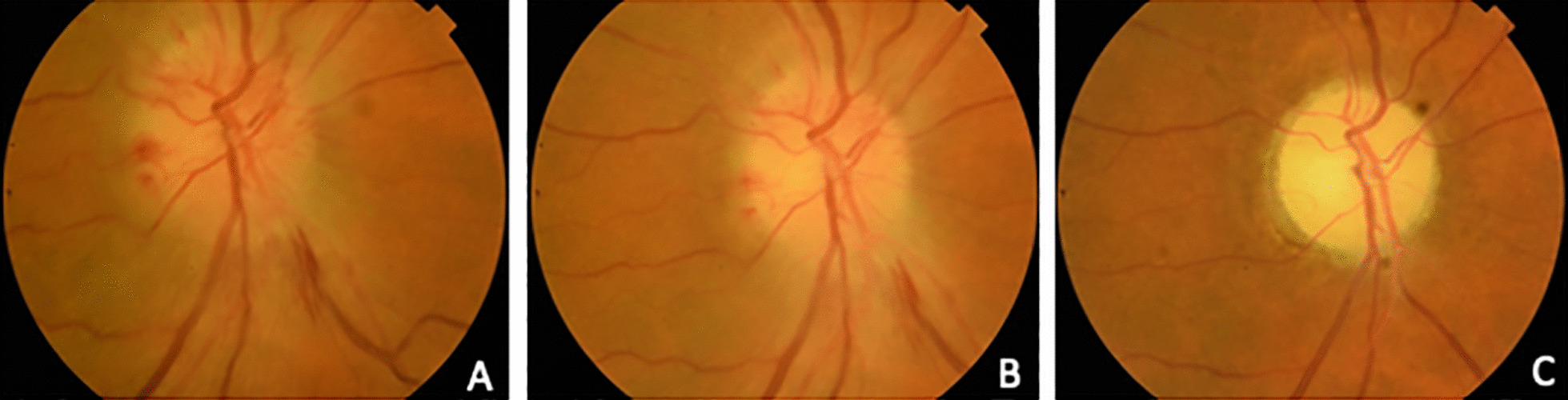
Evolution of the optic nerve head appearance during the follow-up. **A** Optic nerve aspect at baseline; **B** 1 month; and **C** 3 months after the acute event

**Fig. 2:**
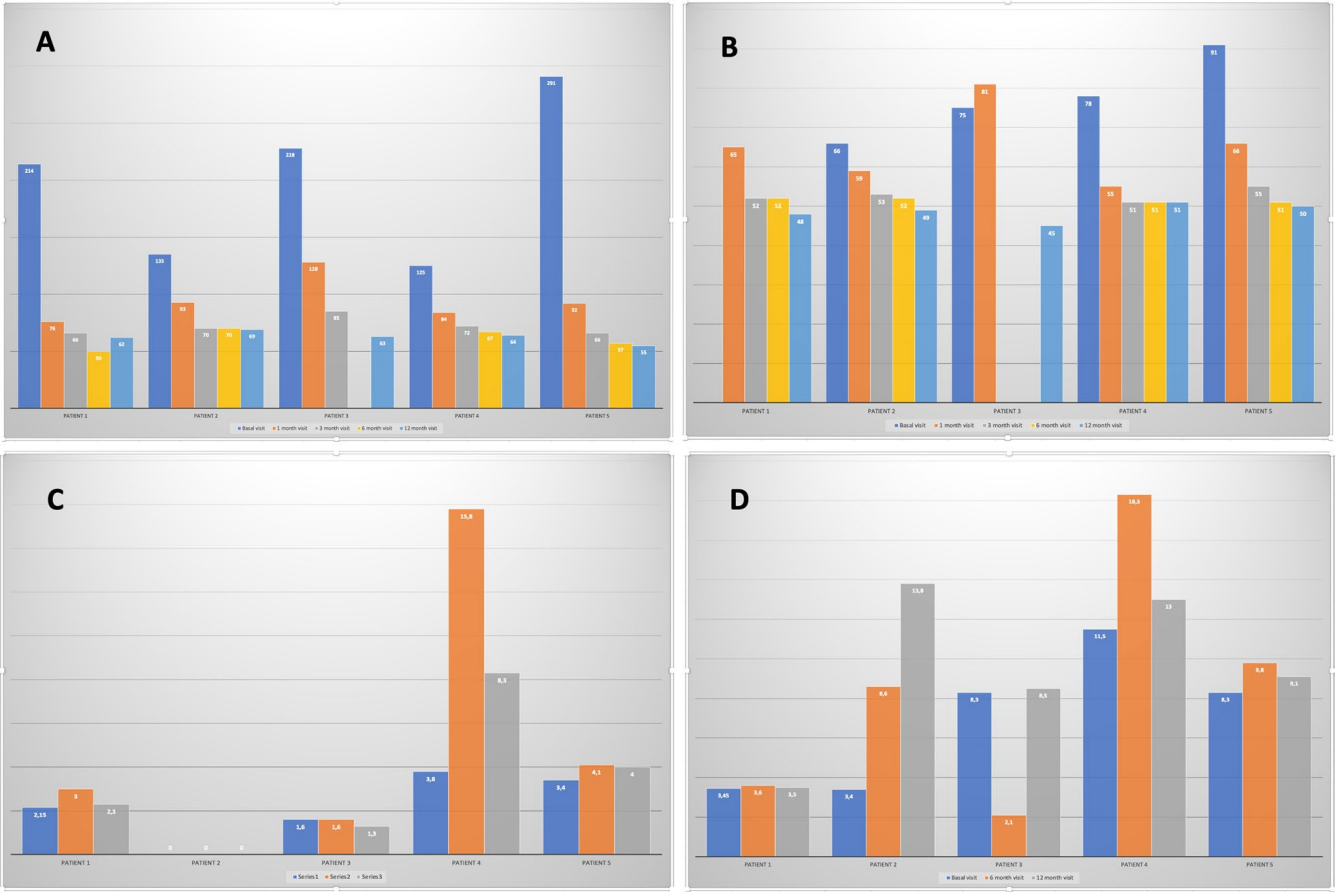
**A** Retinal nerve fiber layer thickness (µm). Reliable data could not be obtained from patient 3 at the last visit. **B** Ganglion cell layer (GCL) thickness (µm). The GCL of patient 1 is missing data. **C** P100 amplitude on the pattern reversal visual-evoked potential with check size of 60 min of arc (PRVEPda60’) measured in microvolts (µV). 0 indicates unrecordable, only noise. **D** P2 amplitude on the flash visual-evoked potential (FVEP) measured in microvolts (µV)

The optic nerve disease was verified by OCT examination. One patient was pseudophakic and four had mild cataracts (C1/N1 according to LOCS III). Lens opacities progressed throughout the follow-up, with a possible relation to the injection procedure, but did not hinder fundus evaluation. The ONH hemorrhages resolved completely and partial atrophy of the ONH was established (Fig. 1). Values of RNFL and GCL thicknesses are shown in Fig. 2; both values decreased over time in all five patients as papillary edema resolved. The remainder of the ocular examination was within normal limits.

**Fig. 3:**
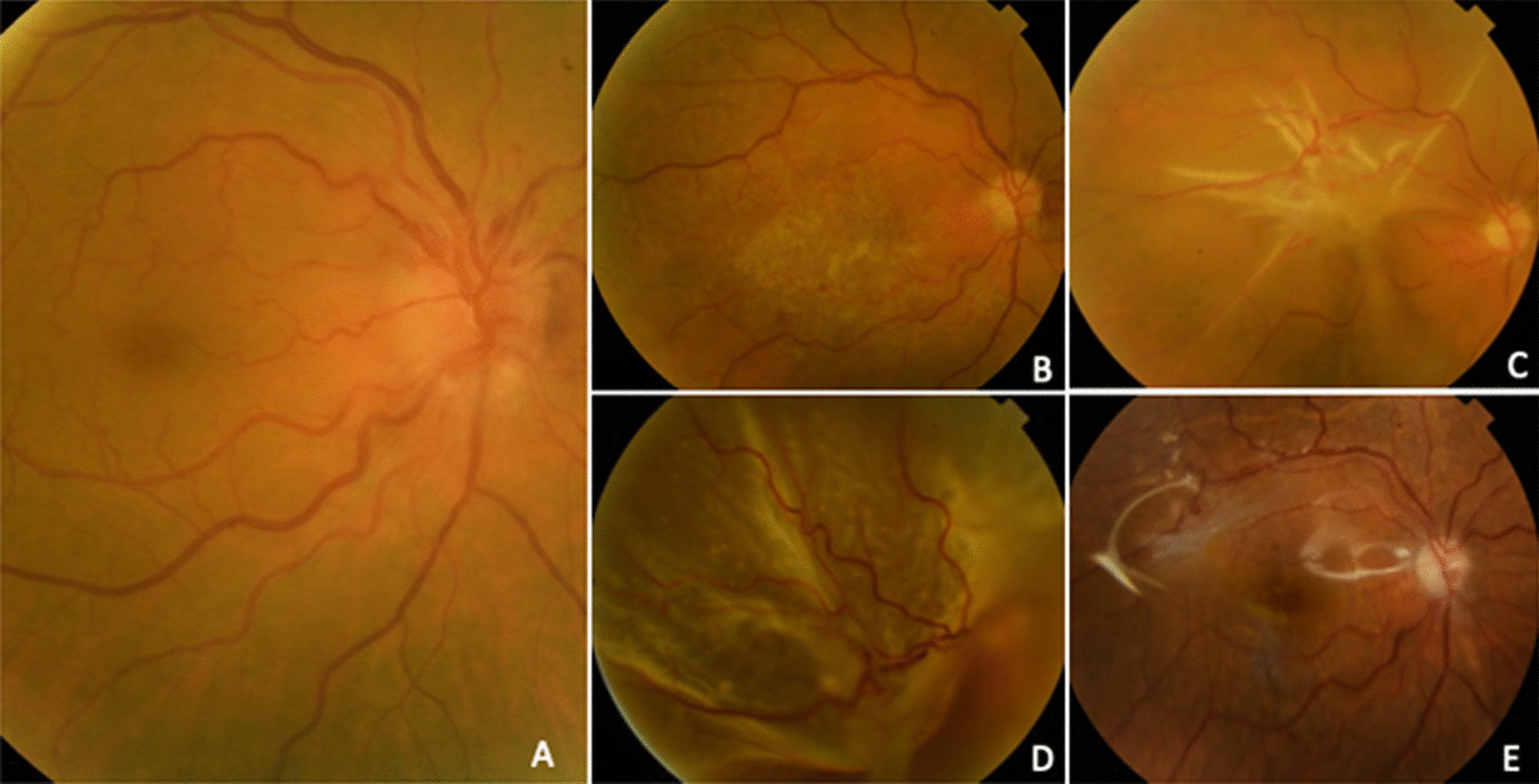
The epiretinal membrane (ERM) in patient 3. **A** Pretreatment; **B** ERM at 1 month; **C** ERM at 3 months; **D** appearance of retinal detachment preoperatively; and **E** postoperatively

The BCVA results at 1, 3, 6 and 12 months are shown in Table 1. Patients 1 and 5 had had NA-AION in their fellow eyes previously with BCVAs of 85 and 11 letters, respectively. Patient 3 developed an epiretinal membrane (ERM) between visits 1 and 3 months leading to a severe decrease in BCVA for which surgery was indicated; however, the patient refused surgery initially and a tractional retinal detachment developed in the posterior pole. The patient finally consented to undergo phacovitrectomy plus silicon oil injection 1 year after ERM diagnosis. A macular hole was intraoperatively detected, which could not be reapplied in surgery due to its large size, but complete anatomic reattachment of the retina was achieved (Fig. 3).

Changes in the P100 amplitude from PRVEP with a check size of 60 min of arc (PRVEPda60´), and P2 amplitude on the FVEP are shown in Fig. 2. Patient 4 had a significant improvement in the P100 amplitude of the PRVEPda60´ at 6 months that decreased at 12 months but stayed above baseline records; patient 3 recording decreased due to the retinal complication; whereas patient 2 had unrecordable results all along the study; and patients 1 and 5 showed moderate improvements at 6-month, also observing a small reduction in patient 1 at 12-month. Besides, patients 2, 4, and 5 showed increased P2 amplitudes on the FVEP; the improvement in patient 2 along the study was especially significant; the great improvement observed in patient 4 at 6 months decreased at 12 months; patients 1’s records barely change after treatment; and although the value decreased in patient 3 while having the tractional retinal detachment at 6-month, the recording recovered after surgery. Results obtained with the check size of 15 min of arc on the PRVEP were unrecordable in most cases at baseline, 6 months, and 12 months.

## Discussion

The current results are encouraging because four patients had improved BCVA; the exception was the patient in whom an ERM developed. Nevertheless, spontaneous improvement is not unusual and recovery of at least three lines of Snellen vision has been reported in up to 40% of patients, although there is some controversy regarding this improvement, as discussed below.

**Fig. 4:**
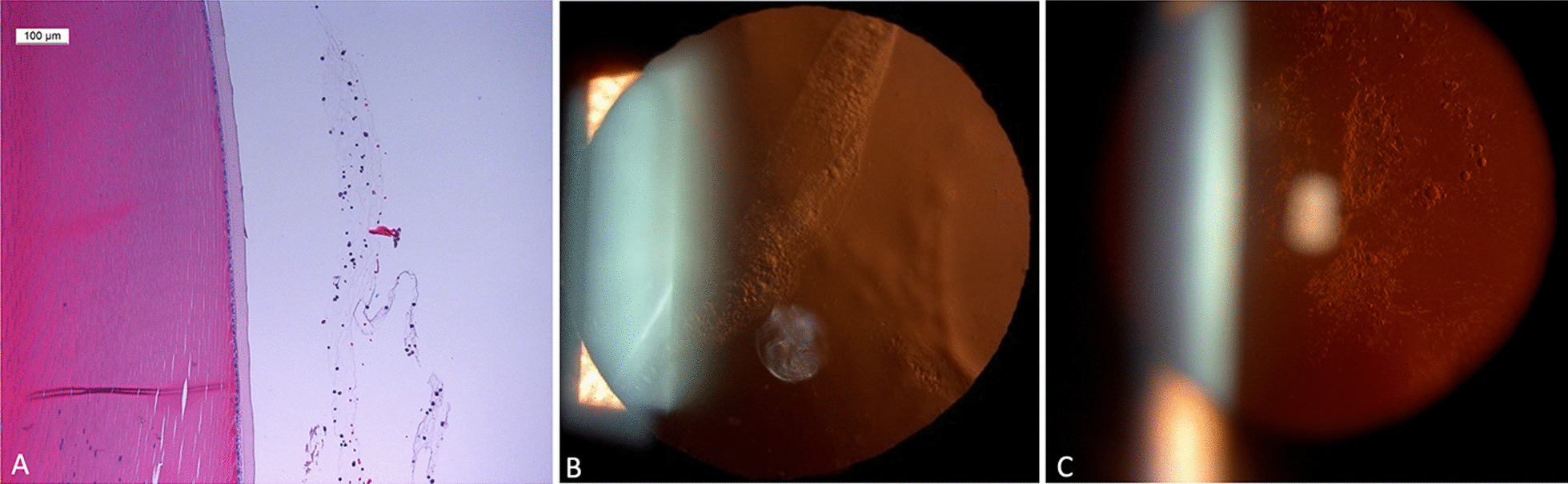
Histologic study of the retrolental aggregates seen in rabbits in **A** our experimental preclinical study. An image of the aggregates in patient 2 in **B** that progressed to significant posterior subcapsular cataract in **C**

It is also important to highlight the improvement of the P100 amplitude on the PRVEPda60´ that seems consistent with the visual results at 6 months. The decrease of the P100 amplitude registries observed in its registry at 12 months in patients 1 and 4 may be due to the cataract progression that especially disturbs this kind of test without affecting that much to the FVEP. Also, the unrecordable registries of PRVEP in patient 2 could be explained considering the appearance of significant lens opacities soon after treatment (Fig. 4), which would prevent the patient from seeing the checkerboard stimulus without affecting the FVEP, as we have explained previously. Following the argument of the influence of lens opacity as the cause of this finding, we should emphasize that patient 5 showed no significant reduction in the amplitude of his PRVEP registries at 12 months probably because he was pseudophakic before treatment.

As expected, the OCT RNFL and GCL thickness measurements showed significant decreases, which agreed with the resolution of the edema and the visible papillary atrophy seen in all patients.

The current study is not the first to offer stem cell therapy for NA-AION. As mentioned, in 2017, the SCOTS group published the results of 10 patients treated with cell therapy and although their results seemed good, with 80% of patients experiencing improvement in the Snellen binocular vision, the study had many design weaknesses [8]. The investigators did not consider a therapeutic window, and in fact, the mean duration of visual loss in the treated eyes was 9.8 years (range, 1–35 years) and it was highly unlikely that axonal recovery would occur after a few weeks. This is a critical point based on experimental data from acute optic nerve injury models showing that, although there is a certain recovery of axonal transportation by week 3, cell death continued or grew in the neurons in which transport through axon had been repaired [29]. Something similar happens in the brain after an ischemic stroke, where neurons in the penumbra area die within days [30]. Another serious problem with the SCOTS study was that the authors used different routes of administration of the BMSCs in highly variable situations, i.e., retrobulbar, sub-Tenons plus intravenously, intra-optic nerve after vitrectomy, and sub-Tenons plus intravenously [8], and it is difficult to understand the possible effects of some of these routes. In addition, the small sample size prevented drawing a conclusion about the real benefits of this treatment.

Recently, the SCOTS group published new results [31] and, despite the improved VA after treatment in 20 of 32 (63%) eyes, their protocol had several problems. The authors included 32 eyes to be treated with BMSC, most of which had age-related macular degeneration (AMD). The number of patients in each category was unclear and included AMD, glaucoma, neovascular glaucoma, and probably diabetic retinopathy, all pathologies whose pathogenesis is unrelated, and no cases of NA-AION were reported in this series. The study also had three treatment arms in which BMSCs were provided via 1, retrobulbar delivery, sub-Tenons, and intravenous (IV) delivery; 2, intravitreal, retrobulbar, sub-Tenons, and IV delivery; and 3, subretinal and IV delivery. In addition, the time since the visual loss was not mentioned, and several ophthalmologists made the follow-up of the patients in a remote way. The study also did not include a discussion regarding the possible mechanisms of action of stem cells via retrobulbar, sub-Tenons, or IV delivery, although the authors suggested a therapeutic option for AMD. We believe that the protocol was too weak to draw conclusions. The same investigators also reported on the treatment of retinitis pigmentosa [32], dominant optic atrophy [33], and other conditions using similar protocols.

The current pilot study was classified by the AEMPS as a phase II CT; thus, its aim was primarily focused on assessing the safety and potential side effects of intravitreal BM-MSCs. Although the improvements in BCVA were striking and detectable, but we should compare these visual changes with that reported in a series of natural evolution. Thus, Raizada and Margolin recently looked at the results of the control group from the Ischemic Optic Neuropathy Decompression Trial comprised of 500 consecutive cases of NA-AION to describe the natural history of the disease [4]. According to that, about 30% of patients would regain ≥ 3 lines of vision at 2 years follow-up and 20% will lose more than 3 lines, but in most patients, the vision will remain unchanged after the resolution of the acute event [5]. Nevertheless, the authors commented that those with a few lines of improvement likely learned to improve their fixation more efficiently without implying a real increase in vision. Besides, the improvement was limited after 6 months and in patients with severe visual loss [34]. While the methods of reporting VA may not be entirely similar between studies, our results, in contrast, showed that our NAION patients initially presenting with a severely impaired VA (Snellen VA of ≤ 20/500 or logMAR BCVA ≤ 1.4) exhibited an improvement after 6 months of at least 1.0 logMAR, except for the patient suffering the retinal complication. Furthermore, at the 12-month marks, 3 of our NAION patients had a logMAR VA ≥ 0.6. Of course, this small series does not allow conclusions to be drawn as our study was designed only to evaluate safety and not efficacy, and a multicenter study is currently recruiting more patients to reassure these results.

When discussing readouts of RNFL and GCL thickness, it is important to note that RNFL measurements obtained closer to the acute episode are increased due to the swelling, thus, GCL changes could be more useful for detecting the structural changes in the first month. In the absence of any therapy improvements in RNFL and GCL thickness would generally not be expected during the natural history of NA-AION as the damaged cells do not spontaneously regenerate substantially, and the overall trend is toward progressive degeneration and atrophy progressive with RNFL loss and thinning observed at 3, 6, and 12 months [35, 36]. In our study, we also observed progressive RNFL loss and thinning at 1, 3, and 6 months after symptom onset. However, beyond that point, the measurements of RNFLT remained stable or showed a lesser degree of decrease compared to the findings of previous studies [35, 36]. We speculate that the relatively lower amount of RNFL loss observed in our NAION patients may contribute to the higher proportion of patients experiencing VA improvement during the recovery stage. Improvements in these readouts typically indicate a positive change in the thickness of these layers, suggesting a potential reversal of damage or preservation of the remaining tissue. However, it is worth noting that spontaneous fluctuations in those measurements in the natural history or individual cases may not necessarily indicate true improvements in visual function or prognosis.

Considering safety, we did not expect to find signs of inflammation, as the work performed by our group in immunocompetent rabbits with the same cells of human origin did not show any sign other than slight inflammation that was related to the injection and resolved after a week [9]. Besides, other studies using this type of cells at the experimental level did not report inflammatory reactions either [37].

The intravitreal injection is straightforward and is often used in cell therapy. The MSCs were injected using a needle through the pars plana into the vitreous cavity. The BM-MSCs stayed in the place where were injected, near of ganglion cells (target cells). These cells did not migrate to subretinal space. For this reason, this route of administration was chosen even though cellular cluster formation of MSC in the vitreous can occur in some cases after intravitreal delivery [38], as it happened to one of our patients who presented a retrolental aggregate, as we had found experimentally that could well be an aggregate of the injected cells [9]. The aggregate disappeared between the 6-month visit and the one-year visit leaving a subcapsular opacification.

Without any doubt, the most serious complication we have had is the development of ERM. Causality assessment in pharmacovigilance implies evaluating the likelihood that a particular treatment causes an observed adverse event. For this reason, we explored several options to determine a possible relationship between the investigational medicinal product and/or the injection procedure and this relevant event. In our case, funduscopy and OCTs before inclusion did not show any sign of ERMs. So, it seems that ERMs might be related to the injection. Several factors may have caused this complication, which, due to its relevance, deserves extensive discussion.

First, the vitreous of that patient visibly adhered to the retina at least partially on OCT at the first visit, and injection probably may have induced a total posterior vitreous detachment (PVD) that together with the presence of BM-MSCs cells could have caused the ERM. This may be a biologically plausible explanation given that PVD is believed to play a critical role in the pathogenesis of idiopathic ERMs, because transient vitreoretinal traction caused by during the development of PVD may cause dehiscence in the internal limited membrane through which glial cells can migrate and proliferate on the inner retinal surface soon after a PVD. In addition, the proliferation and differentiation of hyalocytes located in the vitreous cortical remnants that remain on retinal surface after PVD could induce ERMs [39]. Thus, PVD could have triggered ERM formation.

However, new studies about proteomics have suggested that the growth factors and cytokines are related to the formation of ERM (mainly nerve growth factor, glial cell line-derived growth factor, and transforming growth factor β1). Maybe, MSCs could have been the source of these factors in this case [40, 41]. Thus, the BM-MSC cells injected into the vitreous are another factor to be considered to play a role in the ERM appearance.

Also, in an in vivo experimental model of retinal degeneration in which stem cells were administrated by subretinal and intravitreal route, the authors showed a strong reactive gliosis and reported that deposits of chondroitin sulfate proteoglycans appeared, decreasing cell migration to target tissue [42]. These facts were reported also when other cell types were injected into subretinal space or vitreous [43–47]. Therefore, the development of these complications looks like to be independent of the type of used cells and it is not exclusive for MSCs.

MSCs injected into the vitreous cavity also have been reported to cause ERM and proliferative vitreoretinopathy (PVR) formation and other severe adverse effects such as secondary glaucoma [48–52]. Moreover, MSCs can differentiate to cells like myofibroblasts when they remain on retinal surface. This process could induce fibrosis, PVR, and retinal detachment [52–54]. In addition, MSCs can induce ocular neovascularization, due to their presence increase oxygen request [48, 49]. According to these findings, MSC-Exosome therapy might be safer than cell suspension since cell proliferation is less likely to occur and for this reason, and because of that this type of treatment has been proposed as an alternative to cell therapy [54].

Therefore, the rapid evolution of the ERM observed in our patient leading to tractional retinal detachment may have been related to proliferation, migration, and glial-to-mesenchymal transition in myofibroblasts during ERM progression, as in some experimental studies and treated patients [56]. The patient's refusal to undergo surgery as soon as the membrane was diagnosed may partly justify the poor final result observed.

Considering all what previously said and being a single case, this therapeutic modality can continue to be tested but implementing additional measures to closely monitor the vitreoretinal interface, so that EMRs can be prevented or diagnosed early and treated, to maintain patient’s safety. The risk of inducing ERMs should not be minimized, but given the severity of the underlying process, the lack of therapeutic alternatives, and the good results obtained in this short series, we believe that the risk/benefit ratio still justifies continuing the recruitment of patients, with special consideration of the steps needed for early diagnosis of this possible complication. Adding new inclusion criteria and excluding patients with vitreoretinal adhesion are other measures taken to prevent this complication.

As mentioned, the other interesting finding concerning safety was the presence of a retrolental aggregate in one patient. The traumatic effect on the lens derived from the intravitreal injection was ruled out. We do not know its composition, but in our rabbit experimental studies, the stem cells gathered over the ONH and at the retrolental space with a similar appearance, so we assume it has the same composition. The condensation decreased slightly throughout the follow-up, but a significant posterior subcapsular cataract appeared after 6 months in this patient (Fig. 4).

Interestingly, the appearance of ERM or retrolental aggregates is not mentioned in the series reported by SCOTS group [8, 31].

## Conclusions

In patients with acute NA-AION, intravitreal application of allogeneic bone marrow-derived mesenchymal stem cells appeared to be safe. This clinical trial showed that the injection of BMSCs to treat acute NA-AION was well tolerated. Nevertheless, one patient developed an ERM with bad outcome probably related to the fact that the patient did not agree to be operated on before, and another one showed a transient deposit in the retrolental space which possibly was an aggregate of cells that vanished spontaneously leaving a subcapsular cataract. The positive risk/benefit analysis of this trial motivated the design of a multicenter study that will include more patients that will be performed in four Spanish centers.

### Supplementary Information

**Additional file 1**. TREND flow diagram.

**Additional file 2. Supplementary Table T1.** Follow-up Schedule

## Data Availability

All data generated or analyzed during this study are included in this article.

## Abbreviations

NA-AION: Acute anterior ischemic optic neuropathy
MSV®: Allogeneic bone marrow-derived mesenchymal stem cells
BM-MSCs: Bone marrow mesenchymal stem cells
RNFLT: Retinal nerve fiber layer thicknesses
GCL: Ganglion cell layer thicknesses
SD-OCT: Spectral-domain optical coherence tomography
PRVEP: Pattern-reversal visual-evoked potential
FVEP: Flash visual-evoked potential
