# Supplementary figures and images for: Intravitreal allogeneic mesenchymal stem cells: a non-randomized phase II clinical trial for acute non-arteritic optic neuropathy
Source: Stem Cell Res Ther. 2023 Sep 21;14:261. doi: 10.1186/s13287-023-03500-7 (PMC10512539; doi:10.1186/s13287-023-03500-7)

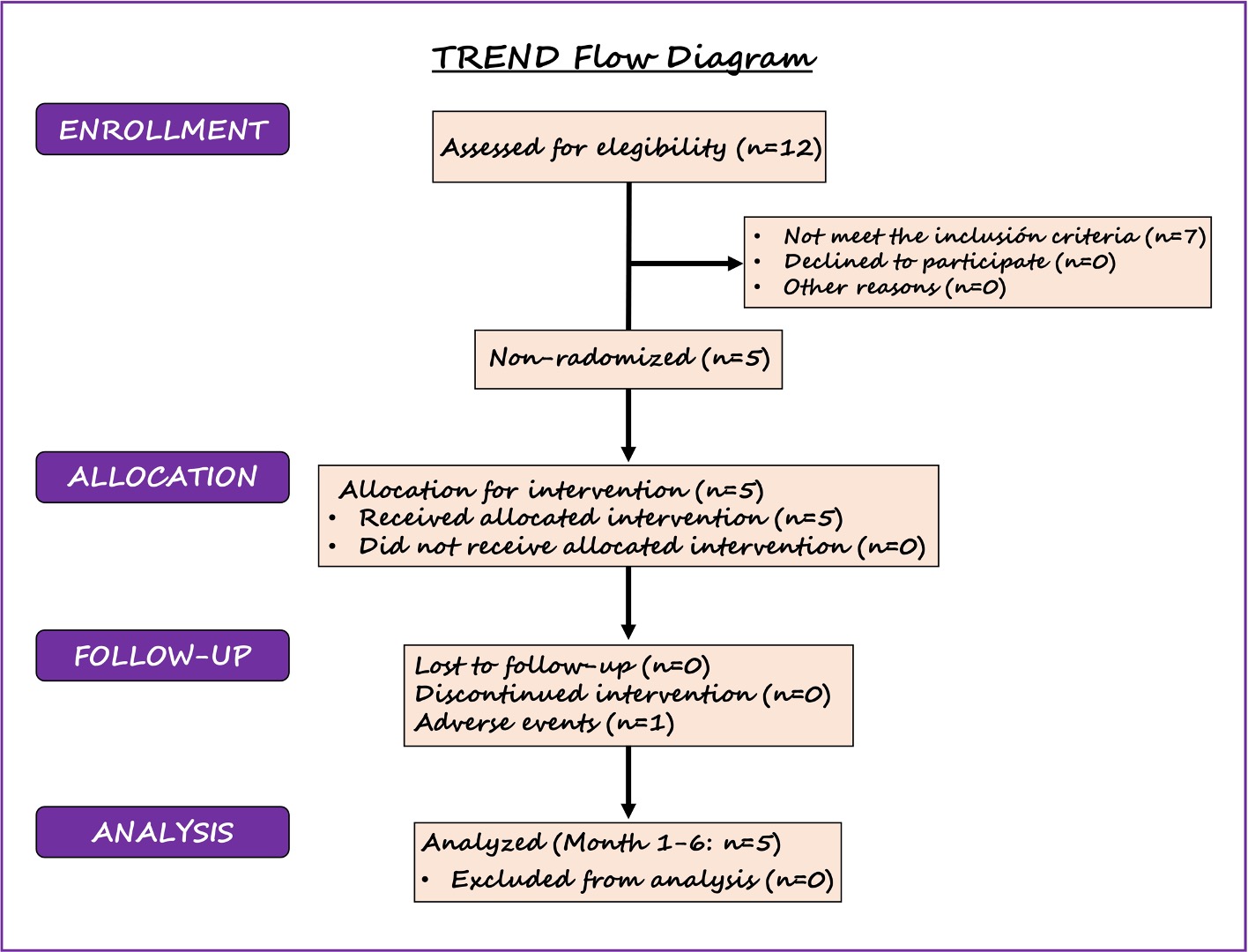

Supplement: Supplementary file 1 — Additional file 1. TREND flow diagram. [file 13287_2023_3500_MOESM1_ESM.jpg]
