# Supplementary material for: Intravitreal allogeneic mesenchymal stem cells: a non-randomized phase II clinical trial for acute non-arteritic optic neuropathy
Source: Stem Cell Res Ther. 2023 Sep 21;14:261. doi: 10.1186/s13287-023-03500-7 (PMC10512539; doi:10.1186/s13287-023-03500-7)
Supplement: Supplementary file 2 — Additional file 2. Supplementary Table T1. Follow-up Schedule [file 13287_2023_3500_MOESM2_ESM.docx]

**Supplementary Table T1. Follow-up Schedule**

**_________________________________________________________________________________________________________________**

|  | **Baseline** | **Visit 0 (TX)** | **Visit 1** | **Visit 2** | **Visit 3** | **Visit 4** | **Visit 5** | **Visit 6** | **NP** | **SP** |
| --- | --- | --- | --- | --- | --- | --- | --- | --- | --- | --- |
| Day |  | BV +7 | V0+1 | V0+7 | V0+30 | V0+90 | V0+180 | V0+360 |  |  |
| Window |  | +2/-4 Days | +/2 Days | +/2 Days | +/2 Days | +/2 Days | +/2 Days | +/2 Days |  |  |
| Informed consent | **✓** |  |  |  |  |  |  |  |  |  |
| Inclusion criteria | **✓** | **✓** |  |  |  |  |  |  |  |  |
| Anamnesis | **✓** | **✓** | **✓** | **✓** | **✓** | **✓** | **✓** | **✓** | **✓** | **✓** |
| Signs and symptoms | **✓** |  |  |  |  |  |  |  |  |  |
| Current diseases | **✓** |  |  |  |  |  |  |  |  |  |
| Concomitant drugs | **✓** | **✓** | **✓** | **✓** | **✓** | **✓** | **✓** | **✓** | **✓** | **✓** |
| BCVA (ETDRS) | **✓** |  |  | **✓** | **✓** | **✓** | **✓** | **✓** | **✓** | **✓** |
| Visual-evoked potentials | **✓** |  |  |  |  |  | **✓** | **✓** |  | **✓** |
| Slit-lamp | **✓** | **✓** | **✓** | **✓** | **✓** | **✓** | **✓** | **✓** | **✓** | **✓** |
| IOP | **✓** | **✓** | **✓** | **✓** | **✓** | **✓** | **✓** | **✓** | **✓** | **✓** |
| Funduscopy | **✓** | **✓** | **✓** | **✓** | **✓** | **✓** | **✓** | **✓** | **✓** | **✓** |
| Retinography | **✓** |  |  |  |  | **✓**** | **✓**** | **✓**** | **✓**** | **✓**** |
| Autofluorescence | **✓** |  |  |  |  | **✓**** | **✓**** | **✓**** | **✓**** | **✓**** |
| Slit-lamp photography |  |  | **✓*** | **✓*** | **✓*** | **✓*** | **✓*** | **✓*** | **✓*** | **✓*** |
| SD-OCT | **✓** |  |  |  | **✓** | **✓** | **✓** | **✓** | **✓** | **✓** |
| ESR, CPR | **✓** |  |  |  |  |  |  |  |  |  |
| Serology | **✓** |  |  |  |  |  |  |  |  |  |
| Treatment |  | **✓** |  |  |  |  |  |  |  |  |
| Safety post-TX |  | **✓** |  |  |  |  |  |  |  |  |
| Adverse events |  | **✓** | **✓** | **✓** | **✓** | **✓** | **✓** | **✓** | **✓** | **✓** |

NP = unscheduled visit; SP = early exit termination; BV = baseline visit; V0 = visit of inclusion; TX = treatment; BCVA = best-corrected visual acuity; ETDRS= Early treatment diabetic retinopathy study optotypes; IOP = intraocular pressure; SD-OCT = spectral-domain optical coherence tomography; ESR = erythrocyte sedimentation rate, CRP = C-reactive protein.
